# Crude probability of death for cancer patients by spread of disease in New South Wales, Australia 1985 to 2014

**DOI:** 10.1002/cam4.3844

**Published:** 2021-05-06

**Authors:** Xue Qin Yu, Paramita Dasgupta, Clare Kahn, Kou Kou, Susanna Cramb, Peter Baade

**Affiliations:** ^1^ Cancer Research Division Cancer Council NSW Sydney Australia; ^2^ Sydney School of Public Health Faculty of Medicine and Health University of Sydney Sydney Australia; ^3^ Cancer Research Centre Cancer Council Queensland Brisbane Queensland Australia; ^4^ Institute of Health and Biomedical Innovation School of Public Health and Social Work Queensland University of Technology Queensland Australia; ^5^ Menzies Health Institute Queensland Griffith University Southport Australia; ^6^ School of Mathematical Sciences Queensland University of Technology Brisbane Australia

**Keywords:** cancer, competing risks, Probability of death, Stage, temporal trends

## Abstract

**Background:**

To estimate trends in the crude probability of death for cancer patients by sex, age and spread of disease over the past 30 years in New South Wales, Australia.

**Methods:**

Population‐based cohort of 716,501 people aged 15–89 years diagnosed with a first primary cancer during 1985–2014 were followed up to 31 December 2015. Flexible parametric relative survival models were used to estimate the age‐specific crude probability of dying from cancer and other causes by calendar year, sex and spread of disease for all solid tumours combined and cancers of the colorectum, lung, female breast, prostate and melanoma.

**Results:**

Estimated 10‐year sex, age and spread‐specific crude probabilities of cancer death generally decreased over time for most cancer types, although the magnitude of the decrease varied. For example, out of 100 fifty‐year old men with localized prostate cancer, 12 would have died from their cancer if diagnosed in 1985 and 3 in 2014. Greater degree of spread was consistently associated with higher probability of dying from cancer, although outcomes for lung cancer were consistently poor. For both males and females, the probability of non‐cancer deaths was higher among older patients, those diagnosed with localized cancers and where cancer survival was higher.

**Conclusion:**

Crude probabilities presented here may be useful in helping clinicians and their patients better understand prognoses and make informed decisions about treatment. They also provide novel insights into the relative contributions that early detection and improved treatments have on the observed temporal patterns in cancer survival.

## Graphical abstract

This study provides a more relevant measure of prognosis for cancer patients ‐ crude probabilities of death due to cancer and other causes. Such information may be useful to clinicians and patients making treatment choices based on cancer type, age and stage at diagnosis.

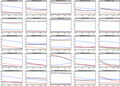

## 1 INTRODUCTION

The population of cancer survivors has steadily increased over past decades in Australia,^1^, ^2^, ^3^, ^4^ due to diagnostic and therapeutic improvements.^5^ As cancer patients survive longer, their risk of dying from causes other than the diagnosed cancer (competing causes) will however continue to increase. Relative survival, although the preferred measure of cancer survival in a population‐based setting,^6^ does not provide a complete picture of patients’ outcomes, as the effects of competing causes of death are ignored. Crude probabilities, however, partition the probability of death from any cause into deaths due to a cancer diagnosis and deaths due to other causes,^7^ thereby quantifying a more real‐world probability of death where cancer patients are also at risk of dying from other (competing) causes. Crude probabilities are thus suggested to be more relevant for risk communication and clinical decision‐making, as they provide key contextual information on the impact of other causes of death among cancer patients.^7^, ^8^

We have previously reported temporal trends in crude probabilities of cancer and other causes of death for a population‐based cohort of Australian cancer patients.^9^ However, that analysis did not consider the effect of spread of disease at diagnosis, a critical factor in cancer patients’ prognoses. Information about spread of disease is not available nationally, but it is recorded in the cancer registry for the state of New South Wales (NSW), the most populous state in Australia. By examining trends in crude probabilities of death for NSW by spread of disease for five major cancer types, this study can provide greater insights into the potential impact of earlier diagnosis and improved treatment on the observed patterns in cancer survival.

## 2 METHODS

Ethics approval was obtained from the NSW Population & Health Services Research Ethics Committee (2016/HRE1203). The NSW Cancer Registry provided approval to access de‐identified data from the Australian Cancer Database, to which all cancer diagnoses in Australia must be legally notified.

Data were extracted for NSW residents aged 15–89 years diagnosed with a first primary tumour between January 1985 and December 2014. Patients’ vital status was obtained through routine annual linkage of cancer records with the Australian National Death Index, with follow‐up to 31 December 2015. Given the unique staging classifications of cancers of the lymphohematopoietic system (ICD‐10: C81‐C96, D45‐D47), we have focussed the analyses on solid cancers only, thus excluding people whose first primary cancer was cancers of the lymphohematopoietic system.

Spread of disease at diagnosis was mainly based on pathology reports and statutory notifications from hospitals, then coded using a modified summary classification as localized (stage I), regional (a combination of stages II and III), distant (stage IV) and unknown (including missing).^10^

Analyses were carried out for all invasive solid cancers combined and the five leading cancer types (Table 1): colorectal, lung, female breast, prostate cancers and melanoma of the skin which together comprised around 66% of all solid cancers diagnosed in NSW during the study period.

**TABLE 1:** Characteristics of the study cohort by spread of disease, NSW, Australia, 1985 to 2014

	Spread of disease (row %)^a^				Overall (col%)
	*Localized*	*Regional*	*Distant*	*Unknown* ^a^	
*Sex*					
Male	170,754 (43)	71,657 (18)	63,341 (16)	87,444 (22)	393,196 (55)
Female	147,511 (46)	84,193 (26)	51,800 (16)	39,801 (12)	323,305 (45)
*Age group at diagnosis (years)*					
15–49	60,520 (55)	25,474 (23)	11,334 (10)	12,861 (12)	110,189 (15)
50–69	149,345 (46)	74,170 (23)	49,674 (15)	49,807 (15)	322,996 (45)
70–89	108,400 (38)	56,206 (20)	54,133 (19)	64,577 (23)	283,316 (40)
*Diagnostic period*					
1985–1994	80,511 (44)	37,561 (20)	30,391 (17)	35,797 (19)	184,260 (26)
1995–2004	99,821 (42)	49,155 (21)	36,258 (15)	50,021 (22)	235,255 (33)
2005–2014	137,933 (47)	69,134 (23)	48,492 (16)	41,427 (14)	296,986 (41)
*Vital status (within 10 years after diagnosis)* ^b^					
Alive	218,833 (60)	76,254 (21)	11,442 (3)	57,033 (16)	363,562 (51)
Dead	99,432 (28)	79,596 (23)	103,699 (29)	70,212 (20)	352,939 (49)
*Cancer (ICD*−*10 codes)* ^c^					
Colorectal (C18‐C20)	31,029 (30)	43,544 (43)	17,282 (17)	9,823 (10)	101,678 (14)
Lung (C33‐C34)	15,487 (21)	13,996 (19)	27,278 (38)	15,621 (22)	72,382 (10)
Melanoma (C43)	67,841 (85)	4,688 (6)	3,717 (5)	3,530 (4)	79,776 (11)
Female breast (C50)	52,689 (51)	35,813 (35)	5,503 (5)	9,646 (9)	103,651 (15)
Prostate (C61)	58,112 (49)	9,683 (8)	6,456 (5)	44,481 (38)	118,732 (17)
All solid tumours^d^	318,265 (44)	155,850 (22)	115,141 (16)	127,245 (18)	716,501 (100)

^a^ Affected during 1993–1999 by a data artefact that caused high proportions of localized cancer cases to be categorised as having unknown degree of spread.

^b^ Not all cases had 10 years of potential follow‐up.

^c^ ICD‐10 International Statistical Classification of Diseases and related health problems, tenth revision.

^d^ Cancers of lymphohematopoietic system (C81‐C96, D45‐D47) were ineligible due to spread of disease information being non‐applicable.

Patients’ survival was measured in days from the date of diagnosis to either the date of death, at 10 years after diagnosis, or the study end date (31 December 2015), whichever came first. Cases alive at the end of follow‐up were censored. Relative survival, a measure of net survival, estimating the chance of surviving assuming that cancer is the only possible cause of death, was used in this study because causes of death recorded in population‐based cancer registries are often inaccurate.^11^ Indeed, the cause of death is not always known, and when known, is not always reliable.^12^, ^13^ Relative survival is calculated as the ratio of the observed survival for the cancer cohort to their expected survival if they were cancer‐free.^6^ Estimates of expected survival were based on all‐cause mortality rates by sex, single year of age and calendar year for NSW.^14^ The cohort method was used as this study was designed to assess temporal trends in survival.^15^

Flexible parametric relative survival models^16^, ^17^ were used for statistical analyses. These models use restricted cubic splines for the baseline cumulative excess hazard to obtain smoothed estimates of the excess mortality rates, allow inclusion of time‐varying effects and continuous covariates, and enable prediction of crude probabilities of death from cancer and other causes from the fitted models.

Two sets of models were fitted for each cancer type: one that included age, year of diagnosis and sex and another that also included spread of disease. The models relating to all solid tumours combined also included a variable representing broad cancer categories by 5‐year survival estimates for Australia to adjust for the case mix.

### 2.1 Model one: Overall crude probabilities of dying

When calculating the overall cancer type‐specific crude probabilities of dying (irrespective of stage), age and year of diagnosis were included as continuous variables using restricted cubic splines (two and four degrees of freedom (df) respectively). Interactions between the spline terms for age and year were also included as previously described.^9^ Where applicable, models were stratified by sex. Likelihood ratio‐tests supported the inclusion of both age and year as time‐dependent effects. Depending on the cancer type, we used 3 to 6 df (2–5 internal ‘knots’) for the baseline, and 2 to 5 df (1–4 internal ‘knots’) for the time‐dependent effects, with the selection of the number of knots in each instance based on minimizing the Bayesian Information Criterion (BIC). All knots were placed at the default positions, for flexible parametric models (See Table 1 in Lambert et al. 2009).^16^ Various studies^9^, ^16^ have previously shown that the measures from flexible parametric models are not sensitive to location or number of knots.

### 2.2 Model two: Crude probabilities of dying by spread of disease

In addition to the variables described above, these models also included spread of disease as a four‐level categorical variable and the second‐order interaction terms between year of diagnosis (restricted cubic spline with two df) and spread of disease. Likelihood ratio tests supported the inclusion of age, year and spread of disease (for all solid cancers combined, prostate and female breast cancer only) as time‐dependent effects. Depending on cancer type, we used 4–6 df (3–5 internal ‘knots’) for the baseline and 2–5 df (1–4 internal ‘knots’) for the time‐dependent effects, with the selection of the number of knots in each instance based on minimizing the BIC. Sensitivity analyses (results not shown) showed strong consistency in the estimated effect sizes regardless of the number of knots.

### 2.3 Estimation of model‐based crude probabilities

Crude probabilities of dying from cancer and other causes were estimated by transforming the fitted model parameters as described by Lambert and colleagues.^7^ Probabilities were predicted until 10 years after diagnosis, consistent with our previous study,^9^ and required extrapolation of the survival functions for patients diagnosed from 2006 onwards. To do this, we used the fitted models to extrapolate the observed survival for the cancer cohort, and utilized life tables based on published actual mortality rates until 2017^14^ and projected mortality rates assuming high life expectancy from 2018 onwards.^18^

Crude probabilities of death were expressed per 100 individuals: that is, how many out of 100 patients diagnosed with cancer in a given year would die from their diagnosed cancer, die from other causes or be alive at 10‐years after diagnosis. Results were tabulated for ages 50, 65 and 80 years for the following years: 1985, 1995, 2005 and 2014.

All analyses were performed with Stata/SE version 16 (StataCorp, TX, USA). The *stpm2 cm* command^7^ was used to predict the crude probability of death and its 95% confidence interval (CI) from the fitted flexible parametric relative survival models. The effect of each term included in the flexible parametric relative survival models was considered to be significant only if *p* < 0.05 (Wald's test, two‐sided).

### 2.4 Sensitivity analyses

Sensitivity analyses for unknown spread of disease were carried out by repeating the crude probability calculations with different assumptions about the true distribution of cases with unknown spread. These assumptions were: A) all such cases were considered to be localized, B) all were considered to be distant, C) all were randomly and equally distributed over the three known categories and D) all were assigned to these three categories based on their survival (e.g. a case who died within 1 year of diagnosis was considered to have distant disease). A case with unknown spread was considered to be localized if they were alive at 10 years follow‐up, and distant if had died within 1 year of diagnosis and regional if had died by end of 10‐year follow‐up but lived for more than 1 year. Of note was the higher proportion of cases with unknown spread of disease during 1993–1999 due to the transition period from the traditional paper notifications to the electronic notification system in the NSW Cancer Registry.^19^

## 3 RESULTS

Of the 730,149 cases who were initially extracted, a total of 13,648 cases were ineligible from the analysis because they were diagnosed on the basis of death certificate only (*n* = 9,209, 1%) or survived for less than 1 day following diagnosis (*n* = 4,439, 0.55%), resulting in a final study cohort of 716,501 cases.

Overall, 44% (*n* = 318,265) of the study cohort were diagnosed with localized disease, 22% (*n* = 155,850) with regional, 16% (*n* = 115,141) with distant and 18% (*n* = 127,245) with unknown spread (Table 1). The proportion of cases with unknown spread for all solid cancers was 22% for males and 12% for females reflecting differences in the mix of cancer types by sex. For individual cancer types, proportions with unknown spread ranged from 4% for melanoma to 38% for prostate cancer and were generally relatively stable over the study period. Key exceptions were prostate cancer, where cases with unknown spread peaked during the 1990 s and early 2000 s, and lung cancer, which showed a similar but less marked pattern in the proportion with unknown spread (Figure S1).

**FIGURE 1:**
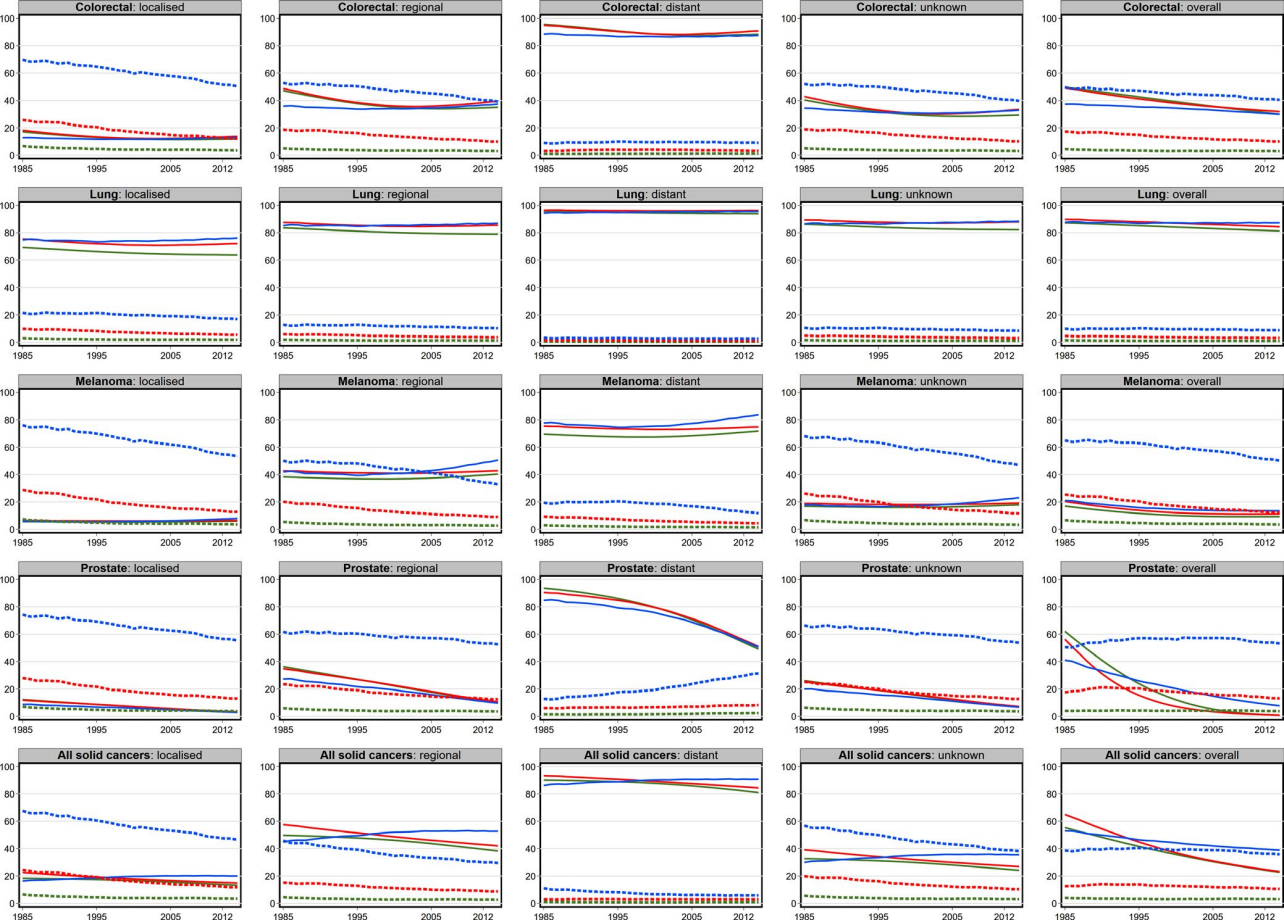
Temporal trends in the predicted 10‐year crude probability of death (per 100 males) due to cancer (solid lines) and other causes (dashed lines) at selected ages by spread of disease for males NSW, Australia, 1985–2014. *The x axis in each graph is the ‘Year of Diagnosis’ and the y axis the ‘Crude probability of death’*. *Dark green represents estimates at age 50*, *red at age 65 and light blue at age 80 years*

While the model including cubic splines for calendar year provided the best fit, interpretation of the year effect is difficult. However, when we included a linear trend for calendar year the coefficient was positive, providing evidence for an increase, and statistically significant for all cancer types (p‐values were all <0.001).

### 3.1 Crude probability of cancer death by spread of disease

For both males and females aged 50 years who were diagnosed with localized or regional disease in 2014, cancer was estimated to be the most common cause of death within the next 10 years. The only exception was for men diagnosed with localized prostate cancer, where non‐cancer causes were the most common cause of death. Most patients were alive 10 years after diagnosis, except for those with lung cancer (Table S1). Patterns were similar for 65‐year‐olds, although for those diagnosed in 2014, the crude probability of dying from other causes was higher than for dying from cancer among both males and females with localized melanoma, females with localized breast or colorectal cancer, and males with non‐distant prostate cancer (Table S2). Increased risk of competing mortality among older patients meant that for both males and females aged 80 years and diagnosed in 2014 with localized melanoma, localized or regional colorectal cancer, the crude probability of dying from cancer was less than the corresponding probability for dying from other causes (Table S3). This was also true for those with both localized and regional prostate and female breast cancers.

### 3.2 Trends in the crude probability of cancer death

**FIGURE 2:**
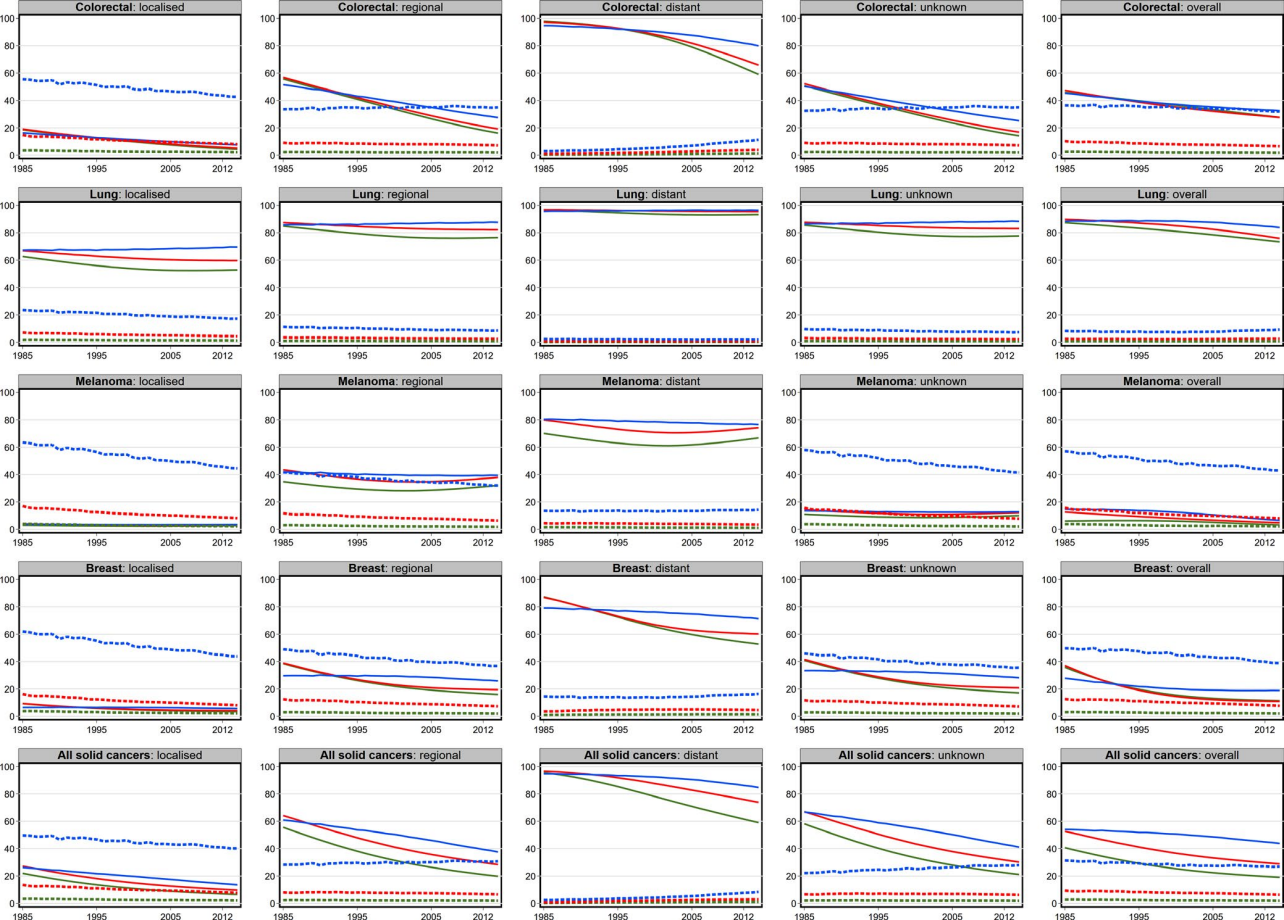
Temporal trends in the predicted 10‐year crude probability of death (per 100 females) due to cancer (solid lines) and other causes (dashed lines) at selected ages by spread of disease for females NSW, Australia, 1985–2014. *The x axis in each graph is the ‘Year of Diagnosis’ and the y axis the ‘Crude probability of death’*. *Dark green represents estimates at age 50*, *red at age 65 and light blue at age 80 years*

When looking at trends in the probability of cancer death by cancer type, the overall trends were not always reflected in the trends by cancer spread. For melanoma in particular, while an overall decrease in the risk of cancer death was seen for all ages in both males and females, within the spread of disease categories there was very little change, and even some slight increases (Figures 1, 2, Tables S1‐S3). These probabilities generally decreased over calendar time for most cancer types, although the magnitude of the decrease varied (Figures 1, 2, Tables S1‐S3). For example, out of 100 fifty‐year‐old men diagnosed with localized prostate cancer in 1985, 12 would have died from their cancer within 10 years, while in 2014 this number was estimated to be only 3. Corresponding numbers for those with regional disease were 36 and 10 respectively, and for those with distant disease were estimated to be 94 and 49. By contrast, the crude probability of dying from cancer remained consistently high for all lung cancer patients, regardless of age or degree of spread.

For some cancer types, while the absolute values varied, overall trends in the probability of dying from cancer were also different across each of the spread of disease categories (Figures 1, 2; Tables S1‐S3). For example, while the overall probability of dying from cancer for patients with melanoma decreased over time for both males and females, within each of the spread of disease categories there was very little change. By contrast, for prostate, female breast and colorectal cancers, the overall decrease was also reflected in the spread specific pattern, apart from males aged 80 years with colorectal cancer. However, even for these cancer types, particularly prostate cancer, the magnitude of the decreasing trend varied within spread categories.

### 3.3 Sensitivity analyses

Sensitivity analyses indicated that the observed trends were robust across the different approaches to classifying cases with unknown spread of disease under various assumptions about their true distribution. As an illustrative example, patterns are shown for prostate and female lung cancers, the two cancer types with the highest percentages of cases with unknown spread of disease in the original study cohort (Figure S2).

## 4 DISCUSSION

In a previous study using national data, we reported that an 80‐year‐old Australian diagnosed with prostate, female breast or colorectal cancers was more likely to die from competing causes within 10 years of diagnosis than die from their cancer.^9^ This study complements that previous work by presenting cancer spread‐specific estimates of crude probabilities of dying for NSW, highlighting the marked variation in the crude probability of dying from cancer by spread of disease and age at diagnosis. In addition, the spread‐specific estimates also varied markedly by cancer type, so that an 80‐year‐old male diagnosed with localized prostate disease in 2014 had a much higher probability of dying from other causes than a similarly aged male with localized lung cancer.

Crude probability estimates as presented here can be easily converted and comprehended in terms of natural frequencies rather than a potentially ambiguous relative risk estimate or survival probability.^8^ Relative risks can exaggerate the perception of a meaningful difference, especially when the absolute risks are low.^20^ They have also been shown to be harder to understand and to be more often interpreted incorrectly by the general public than absolute measures.^20^, ^21^ Also, by incorporating competing mortality, crude probabilities portray the real‐world impact of a cancer diagnosis on prognosis,^8^, ^22^ something the widely used relative survival measure does not do. In summary, these measures complement the typical reporting of cancer survival measures such as relative survival, thus providing clinicians and cancer patients with a more complete picture of the prognosis after a diagnosis of cancer.

By considering competing causes of death, population‐based estimates of crude probabilities of cancer death provide contextualized information which may be of use to clinicians and patients making treatment choices based on cancer type, age at diagnosis and spread of disease.^22^ For younger patients, and those with distant spread disease or lung cancer, the risk of dying from cancer would reasonably be the primary consideration. In contrast, for older patients diagnosed with early stage disease (except for lung cancer) who were probably more likely to die from competing causes, less aggressive cancer treatments with potentially lower long‐term side effects may be more appropriate.

Results on temporal trends in survival must be interpreted with care,^23^ because while increases in cancer survival could be attributed to improved treatment, or to greater availability of diagnostic facilities among other prognostic factors, they can also be due to statistical artefacts. One such statistical artefact is stage migration,^24^ a shift with time in the stage distribution of a cancer towards apparently higher stage disease because of more complete identification of disease spread in more recent periods. In addition, that our data on spread of cancer are incomplete and varied with time prevented us from being able to precisely separating the contributions of early detection and treatment to the observed temporal spread‐specific trends. However, while we cannot rule out entirely the impact of stage migration and incomplete data on spread of cancer on our observed temporal trends, looking at spread‐specific trends and overall trends (all levels of spread combined) it is possible to provide some insights into contributions of early detection and treatment to the observed trends. For example, for melanoma in males, the overall decrease in cancer deaths over time, while the spread‐specific trends were constant may suggest that there is little treatment/management effect, but there was a diagnostic effect. In contrast, for colorectal cancer, there was a decrease overall, but also decreases in each spread category – so even though there may be a diagnostic effect, there is also evidence of a management effect.

Notwithstanding these considerations, the patterns reported here offer novel insights into the survival status of Australian cancer patients and how these measures have changed over time. For both males and females across all age groups and level of disease spread, the crude probability of dying from cancer decreased over the 30 years for all solid tumours combined, although this decrease was more pronounced for younger patients, and those with regional spread, reflecting the higher competing mortality for older patients. These patterns probably reflect improved treatments over time and/or the long‐term results of effective cancer screening programs, especially for breast cancer,^25^ where the likelihood of dying from cancer declined for women aged 80 years as well as the screening age groups (50 and 65 years). For prostate cancer, screening with prostate‐specific antigen testing increases both earlier diagnosis and detection of smaller and slower growing tumours, so the introduction of such testing would be expected to artificially increase estimated survival and change the crude probability of dying from cancer.^26^ However, evidence of improved survival coincident with declining mortality for those with localized and regional disease^27^ suggests additional survival benefits driven by improved treatments. In contrast, the consistently high probability of dying from lung cancer over the study period, regardless of age and spread of disease highlights the limited advances in lung cancer treatment^1^ and the current lack of any screening program.

There is evidence that cardiovascular disease (CVD) is a leading cause of competing mortality among cancer patients both in Australia^28^ and internationally.^29^, ^30^, ^31^ As well as reflecting the high mortality burden in the general Australian population,^14^ it may also at least partially reflect the increased risks of treatment‐related CVD among cancer survivors, particularly for older patients.^32^ Although it is beyond the scope of this study to identify the specific causes of non‐cancer deaths among our cohort, multidisciplinary care, promotion of healthy lifestyle choices and regular monitoring of high‐risk people, should be a priority in the long‐term care of all cancer survivors.^29^, ^30^

Some limitations to this study include lack of data on treatment and comorbidities. Also, the extrapolation of 10‐year probabilities of dying for more recently diagnosed patients assumed that the effect of calendar time will remain the same in future years. As such, estimates for those diagnosed in 2014 may need to be interpreted more cautiously. However, a previous study^33^ suggests that the extrapolation from flexible parametric models is robust, especially when at least some of the cohort had complete follow‐up, as is the case for this current study. Also, the data for spread of cancer at diagnosis were not complete, particularly for prostate cancer, which mean the spread‐specific estimates of the probabilities of dying from cancer are underestimated. In addition, we are unable to carry out multiple imputation on the incomplete data on spread of cancer at diagnosis due to a lack of additional relevant clinical information, which are predictive of missing spread data, such as morphology, grade or tumour size as well as treatment data.^34^, ^35^ Further, while it is possible to use multiple imputation within the flexible parametric survival model framework used in this study, it is not possible to apply the postestimation command stpm2 cm within that multiple imputation. As a result of this, we used sensitivity analyses on unknown spread. Although the assumptions regarding unknown spread are subjective, the results from these sensitivity analyses suggested that these observed trends may be reliable. This is also supported by a recent assessment of this variable, which suggested that survival analyses stratified by the known‐spread categories may be sufficient for major cancer types such as colorectal or lung cancer.^36^ Finally, interpretation of the reasons for the observed temporal patterns in spread‐specific estimated probability of dying from cancer should be made cautiously, as both lead‐time bias (advancing the date of diagnosis without postponing the time of death) and length bias (detection of slower growing tumours that would not otherwise have been diagnosed or have caused death)^25^ may have impacted estimates. We have therefore provided data on the trends for unknown spread of disease as well as overall estimates of the crude probability to aid the interpretation of the spread‐specific patterns.

Study strengths include the use of a large population‐based cohort over a 30‐year time span, and inclusion of a wide range of individual cancer types with high (female breast, prostate and melanoma), moderate (colorectal) and poor survival (lung). Also, survival was analysed in a framework of relative survival, meaning that definitive cause of death information, which can be problematic in cancer registry data,^11^ was not required.

## 5 CONCLUSION

By reporting trends in the crude probabilities of cancer patients dying from cancer, dying from other causes, or being alive 10 years after diagnosis stratified by spread of disease, these results can provide important insights to aid the discussion of cancer outcomes and treatment decisions. The reported changes over time in the spread‐ and cancer type‐specific probabilities suggests probable differential roles for early detection and improved treatment on cancer outcomes.

## CONFLICT OF INTEREST

None declared.

## AUTHOR CONTRIBUTIONS

Yu XQ: Conceptualization, Investigation, Methodology, Writing–original draft and Writing ‐ review and editing. Dasgupta P: Conceptualization, Data curation, Formal analyses, Investigation, Methodology, Writing ‐ review and editing. Kahn C: Writing ‐ review and editing. Kou K: Methodology, Writing ‐ review and editing. Cramb S: Methodology, Writing ‐ review and editing. Baade P: Conceptualization, Investigation, Methodology, Supervision, Writing ‐ review and editing.

## ETHICAL APPROVAL

Ethics approval was obtained from the NSW Population & Health Services Research Ethics Committee (2016/HRE1203). The NSW Cancer Registry provided approval to access de‐identified dta from the Australian Cancer Database, to which all cancer diagnoses in Australia must be alegally notified.

## Supporting information

Supplementary Material

## Data Availability

The data that support the findings of this study are available from NSW Cancer Institute. Restrictions apply to the availability of these data, which were used under license for this study. Data are available at <https://www.cancer.nsw.gov.au/research‐and‐data/cancer‐data‐and‐statistics/request‐linked‐unit‐record‐data‐for‐research>with the permission of NSW Cancer Institute.

## References

[1] Cancer survival and prevalence in Australia . Period estimates from 1982 to 2010. Asia Pac J Clin Oncol. 2013;9:29‐39. 23418847 10.1111/ajco.12062

[2] Yu XQ , Clements M , O'Connell D . Projections of cancer prevalence by phase of care: a potential tool for planning future health service needs. J Cancer Surviv. 2013;7:641‐651. 23921991 10.1007/s11764-013-0303-9

[3] Yu XQ , De Angelis R , Luo Q , Kahn C , Houssami N , O'Connell DL . A population‐based study of breast cancer prevalence in Australia: predicting the future health care needs of women living with breast cancer. BMC Cancer. 2014;14:936. 25494610 10.1186/1471-2407-14-936 PMC4295409

[4] Yu XQ , Luo Q , Smith DP , Clements MS , O'Connell DL . Prostate cancer prevalence in New South Wales Australia: a population‐based study. Cancer Epidemiol. 2015;39:29‐36. 25530551 10.1016/j.canep.2014.11.009

[5] Arnold M , Rutherford MJ , Bardot A , et al. Progress in cancer survival, mortality, and incidence in seven high‐income countries 1995–2014 (ICBP SURVMARK‐2): a population‐based study. Lancet Oncol. 2019;20:1493‐1505. 31521509 10.1016/S1470-2045(19)30456-5 PMC6838671

[6] Ederer F , Axtell LM , Cutler SJ . The relative survival rate: a statistical methodology. Natl Cancer Inst Monogr. 1961;6:101‐121. 13889176

[7] Lambert PC , Dickman PW , Nelson CP , Royston P . Estimating the crude probability of death due to cancer and other causes using relative survival models. Stat Med. 2010;29:885‐895. 20213719 10.1002/sim.3762

[8] Eloranta S , Adolfsson J , Lambert PC , et al. How can we make cancer survival statistics more useful for patients and clinicians: an illustration using localized prostate cancer in Sweden. Cancer Causes Control. 2013;24:505‐515. 23296456 10.1007/s10552-012-0141-5

[9] Dasgupta P , Cramb S , Kou K , Yu XQ , Baade PD . Temporal trends in net and crude probability of death from cancer and other causes in the Australian population, 1984–2013. Cancer Epidemiol. 2019;62:1984‐2013. 10.1016/j.canep.2019.101568 31330423

[10] Yu XQ , O'Connell DL , Gibberd RW , Armstrong BK . Assessing the impact of socio‐economic status on cancer survival in New South Wales, Australia 1996–2001. Cancer Causes Control. 2008;19:1383‐1390. 18704715 10.1007/s10552-008-9210-1

[11] Percy C , Stanek E 3rd , Gloeckler L . Accuracy of cancer death certificates and its effect on cancer mortality statistics. Am J Public Health. 1981;71:242‐250. 7468855 10.2105/ajph.71.3.242 PMC1619811

[12] Hinchliffe SR , Abrams KR , Lambert PC . The impact of under and over‐recording of cancer on death certificates in a competing risks analysis: a simulation study. Cancer Epidemiol. 2013;37:11‐19. 22999870 10.1016/j.canep.2012.08.012

[13] Lloyd‐Jones DM , Martin DO , Larson MG , Levy D . Accuracy of death certificates for coding coronary heart disease as the cause of death. Ann Intern Med. 1998;129:1020‐1026. 9867756 10.7326/0003-4819-129-12-199812150-00005

[14] Australian Bureau of Statistics. 3302.0 ‐ Deaths . Australia, 2017. Available from URL: http://www.abs.gov.au/AUSSTATS/abs@.nsf/DetailsPage/3302.02017?OpenDocument [accessed 10 September 2019.

[15] Brenner H , Gefeller O , Hakulinen T . Period analysis for ‘up‐to‐date’ cancer survival data: theory, empirical evaluation, computational realisation and applications. Eur J Cancer. 2004;40:326‐335. 14746849 10.1016/j.ejca.2003.10.013

[16] Lambert PC , Royston P . Further development of flexible parametric models for survival analysis. The Stata Journal. 2009;9:265‐290.

[17] Nelson CP , Lambert PC , Squire IB , Jones DR . Flexible parametric models for relative survival, with application in coronary heart disease. Stat Med. 2007;26:5486‐5498. 17893893 10.1002/sim.3064

[18] Australian Bureau of Statistic. 3222.0 ‐ Population Projections . Australia, 2017 (base) to 2066. Available from URL: http://www.abs.gov.au/AUSSTATS/abs@.nsf/allprimarymainfeatures/5A9C0859C5F50C30CA25718C0015182F?opendocument [accessed 1 December 2018.

[19] Barraclough H , Morrell S , Arcorace M , McElroy HJ , Baker DF . Degree‐of‐spread artefact in the New South Wales Central Cancer Registry. Aust N Z J Public Health. 2008;32:414‐416. 18959542 10.1111/j.1753-6405.2008.00271.x

[20] Fagerlin A , Zikmund‐Fisher BJ , Ubel PA . Helping patients decide: ten steps to better risk communication. J Natl Cancer Inst. 2011;103:1436‐1443. 21931068 10.1093/jnci/djr318 PMC3218625

[21] Freeman ALJ . How to communicate evidence to patients. Drug Ther Bull. 2019;57:119‐124. 31345957 10.1136/dtb.2019.000008 PMC6678057

[22] Howlader N , Mariotto AB , Woloshin S , Schwartz LM . Providing clinicians and patients with actual prognosis: cancer in the context of competing causes of death. J Natl Cancer Inst Monogr. 2014;2014:255‐264. 25417239 10.1093/jncimonographs/lgu022 PMC4841170

[23] Dickman PW , Adami HO . Interpreting trends in cancer patient survival. J Intern Med. 2006;260:103‐117. 16882274 10.1111/j.1365-2796.2006.01677.x

[24] Feinstein AR , Sosin DM , Wells CK . The Will Rogers phenomenon. Stage migration and new diagnostic techniques as a source of misleading statistics for survival in cancer. N Engl J Med. 1985;312:1604‐1608. 4000199 10.1056/NEJM198506203122504

[25] Yu XQ , O'Connell DL , Gibberd RW , Coates AS , Armstrong BK . Trends in survival and excess risk of death after diagnosis of cancer in 1980–1996 in New South Wales. Australia. Int J Cancer. 2006;119:894‐900. 16550595 10.1002/ijc.21909

[26] Charvat H , Bossard N , Daubisse L , Binder F , Belot A , Remontet L . Probabilities of dying from cancer and other causes in French cancer patients based on an unbiased estimator of net survival: a study of five common cancers. Cancer Epidemiol. 2013;37:857‐863. 24063904 10.1016/j.canep.2013.08.006

[27] Feletto E , Bang A , Cole‐Clark D , Chalasani V , Rasiah K , Smith DP . An examination of prostate cancer trends in Australia, England, Canada and USA: Is the Australian death rate too high? World J Urol. 2015;33:1677‐1687. 25698456 10.1007/s00345-015-1514-7 PMC4617845

[28] Ye Y , Otahal P , Marwick TH , Wills KE , Neil AL , Venn AJ . Cardiovascular and other competing causes of death among patients with cancer from 2006 to 2015: an Australian population‐based study. Cancer. 2019;125:442‐452. 30311655 10.1002/cncr.31806

[29] Strongman H , Gadd S , Matthews A , et al. Medium and long‐term risks of specific cardiovascular diseases in survivors of 20 adult cancers: a population‐based cohort study using multiple linked UK electronic health records databases. Lancet. 2019;394:1041‐1054. 31443926 10.1016/S0140-6736(19)31674-5 PMC6857444

[30] Sturgeon KM , Deng L , Bluethmann SM , et al. A population‐based study of cardiovascular disease mortality risk in US cancer patients. Eur Heart J. 2019;40:3889‐3897. 31761945 10.1093/eurheartj/ehz766 PMC6925383

[31] Afifi AM , Saad AM , Al‐Husseini MJ , Elmehrath AO , Northfelt DW , Sonbol MB . Causes of death after breast cancer diagnosis: a US population‐based analysis. Cancer. 2020;126:1559‐1567. 31840240 10.1002/cncr.32648

[32] Pierre‐Victor D , Pinsky PF , McCaskill‐Stevens W . Other‐ and all‐cause Mortality among women with breast cancer. Cancer Epidemiol. 2020;65:101694 32135504 10.1016/j.canep.2020.101694

[33] Andersson TM , Dickman PW , Eloranta S , Lambe M , Lambert PC . Estimating the loss in expectation of life due to cancer using flexible parametric survival models. Stat Med. 2013;32:5286‐5300. 24038155 10.1002/sim.5943

[34] Nur U , Shack LG , Rachet B , Carpenter JR , Coleman MP . Modelling relative survival in the presence of incomplete data: a tutorial. Int J Epidemiol. 2010;39:118‐128. 19858106 10.1093/ije/dyp309

[35] Sterne JAC , White IR , Carlin JB , et al. Multiple imputation for missing data in epidemiological and clinical research: potential and pitfalls. BMJ. 2009;338:b2393. 19564179 10.1136/bmj.b2393 PMC2714692

[36] Lawrance S , Bui C , Mahindra V , Arcorace M , Cooke‐Yarborough C . Assessing a modified‐AJCC TNM staging system in the New South Wales Cancer Registry. Australia. BMC Cancer. 2019;19:850. 31462255 10.1186/s12885-019-6062-x PMC6714314

