# Supplementary material for: Crude probability of death for cancer patients by spread of disease in New South Wales, Australia 1985 to 2014
Source: Cancer Med. 2021 May 6;10(11):3524–32. doi: 10.1002/cam4.3844 (PMC8178481; doi:10.1002/cam4.3844)
Supplement: Supplementary file 1 — Supplementary Material [file CAM4-10-3524-s001.docx]

**Supplemental Table S1** Predicted crude probabilities of cancer, other cause deaths and being alive (per 100 persons), at 10 years after diagnosis by sex, spread of disease and period, 1985-2014, at age 50 years.

| ***Crude probabilities (Of 100 cancer patients, how many would have each status at 10-years follow-up)^1^*** | | | | | | | | | | | | | |
| --- | --- | --- | --- | --- | --- | --- | --- | --- | --- | --- | --- | --- | --- |
|  |  | Death due to cancer | | | | Death from other causes | | | | Alive | | | |
| Cancer | Spread of disease | *Year of diagnosis* | | | | *Year of diagnosis* | | | | *Year of diagnosis* | | | |
|  |  | *1985* | *1995* | *2005* | *2014* | *1985* | *1995* | *2005* | *2014* | *1985* | *1995* | *2005* | *2014* |
| ***MALES*** | | | | | | | | | | | | | |
| Colorectal | Overall^2^ | 50 | 43 | 36 | 30 | 5 | 3 | 3 | 3 | 45 | 54 | 61 | 67 |
|  | Localised^3^ | 17 | 13 | 12 | 12 | 7 | 5 | 4 | 4 | 76 | 82 | 84 | 84 |
|  | Regional | 47 | 38 | 34 | 35 | 5 | 4 | 3 | 3 | 48 | 58 | 63 | 62 |
|  | Distant | 96 | 91 | 88 | 88 | 1 | 1 | 1 | 1 | 3 | 8 | 11 | 11 |
|  | Unknown | 40 | 32 | 28 | 29 | 5 | 4 | 3 | 3 | 55 | 64 | 69 | 68 |
| Lung | Overall | 87 | 85 | 83 | 81 | 1 | 1 | 1 | 1 | 12 | 14 | 16 | 18 |
|  | Localised^3^ | 69 | 66 | 64 | 64 | 3 | 2 | 2 | 2 | 28 | 32 | 34 | 34 |
|  | Regional | 84 | 81 | 80 | 79 | 2 | 1 | 1 | 1 | 14 | 18 | 19 | 20 |
|  | Distant | 96 | 95 | 94 | 94 | 1 | 0 | 0 | 1 | 3 | 5 | 6 | 5 |
|  | Unknown | 86 | 84 | 83 | 82 | 2 | 1 | 1 | 1 | 12 | 15 | 16 | 17 |
| Melanoma | Overall | 17 | 12 | 9 | 9 | 7 | 5 | 4 | 4 | 76 | 83 | 87 | 87 |
|  | Localised^3^ | 6 | 5 | 6 | 6 | 7 | 5 | 4 | 4 | 87 | 90 | 90 | 90 |
|  | Regional | 38 | 37 | 37 | 40 | 6 | 4 | 3 | 3 | 56 | 59 | 60 | 57 |
|  | Distant | 69 | 68 | 68 | 72 | 3 | 2 | 2 | 1 | 28 | 30 | 30 | 27 |
|  | Unknown | 17 | 16 | 16 | 18 | 7 | 5 | 4 | 3 | 76 | 79 | 80 | 79 |
| Prostate | Overall | 62 | 24 | 5 | 2 | 4 | 4 | 4 | 4 | 34 | 72 | 91 | 94 |
|  | Localised^3^ | 12 | 9 | 5 | 3 | 7 | 5 | 4 | 4 | 81 | 86 | 91 | 93 |
|  | Regional | 36 | 27 | 18 | 10 | 6 | 4 | 4 | 4 | 58 | 69 | 78 | 86 |
|  | Distant | 94 | 86 | 71 | 49 | 1 | 1 | 2 | 2 | 5 | 13 | 27 | 49 |
|  | Unknown | 26 | 19 | 12 | 7 | 6 | 4 | 4 | 4 | 68 | 77 | 84 | 89 |
| All solid | Overall | 55 | 42 | 30 | 23 | 4 | 3 | 3 | 3 | 41 | 55 | 67 | 74 |
|  | Localised^3^ | 18 | 17 | 16 | 13 | 7 | 4 | 4 | 3 | 75 | 79 | 80 | 84 |
|  | Regional | 50 | 48 | 44 | 38 | 5 | 3 | 3 | 3 | 45 | 49 | 53 | 59 |
|  | Distant | 90 | 89 | 86 | 81 | 1 | 1 | 1 | 1 | 9 | 10 | 13 | 18 |
|  | Unknown | 33 | 31 | 28 | 24 | 6 | 4 | 3 | 3 | 61 | 65 | 69 | 73 |
| ***FEMALES*** | | | | | | | | | | | | | |
| Colorectal | Overall | 46 | 40 | 33 | 28 | 3 | 2 | 2 | 2 | 51 | 58 | 65 | 70 |
|  | Localised^3^ | 19 | 12 | 8 | 4 | 3 | 3 | 2 | 2 | 78 | 85 | 90 | 94 |
|  | Regional | 56 | 41 | 27 | 16 | 2 | 2 | 2 | 2 | 42 | 57 | 71 | 82 |
|  | Distant | 98 | 93 | 79 | 59 | 0 | 1 | 1 | 1 | 2 | 6 | 20 | 40 |
|  | Unknown | 51 | 37 | 24 | 14 | 2 | 2 | 2 | 2 | 47 | 61 | 74 | 84 |
| Lung | Overall | 87 | 83 | 79 | 73 | 1 | 1 | 1 | 1 | 12 | 16 | 20 | 26 |
|  | Localised^3^ | 63 | 56 | 53 | 53 | 2 | 2 | 1 | 1 | 35 | 42 | 46 | 46 |
|  | Regional | 85 | 79 | 76 | 77 | 1 | 1 | 1 | 1 | 14 | 20 | 23 | 22 |
|  | Distant | 97 | 95 | 93 | 93 | 0 | 0 | 0 | 0 | 3 | 5 | 7 | 7 |
|  | Unknown | 86 | 80 | 77 | 78 | 1 | 1 | 1 | 1 | 13 | 19 | 22 | 21 |
| Melanoma | Overall | 6 | 6 | 5 | 3 | 4 | 3 | 3 | 2 | 90 | 91 | 92 | 95 |
|  | Localised^3^ | 3 | 2 | 2 | 3 | 4 | 3 | 3 | 2 | 93 | 95 | 95 | 95 |
|  | Regional | 35 | 29 | 29 | 32 | 3 | 3 | 2 | 2 | 62 | 68 | 69 | 66 |
|  | Distant | 70 | 63 | 62 | 67 | 2 | 1 | 1 | 1 | 28 | 36 | 37 | 32 |
|  | Unknown | 11 | 9 | 9 | 10 | 4 | 3 | 2 | 2 | 85 | 88 | 89 | 88 |
| Breast | Overall | 36 | 20 | 13 | 11 | 3 | 3 | 2 | 2 | 61 | 77 | 85 | 87 |
|  | Localised^3^ | 9 | 6 | 4 | 3 | 4 | 3 | 3 | 2 | 87 | 91 | 93 | 95 |
|  | Regional | 39 | 26 | 19 | 16 | 3 | 3 | 2 | 2 | 58 | 71 | 79 | 82 |
|  | Distant | 87 | 73 | 60 | 53 | 1 | 1 | 1 | 1 | 12 | 26 | 39 | 46 |
|  | Unknown | 41 | 28 | 21 | 17 | 3 | 3 | 2 | 2 | 56 | 69 | 77 | 81 |
| All solid | Overall | 41 | 29 | 22 | 19 | 3 | 3 | 2 | 2 | 56 | 68 | 76 | 79 |
|  | Localised^3^ | 22 | 14 | 9 | 7 | 3 | 3 | 2 | 2 | 75 | 83 | 89 | 91 |
|  | Regional | 56 | 38 | 26 | 20 | 2 | 2 | 2 | 2 | 42 | 60 | 72 | 78 |
|  | Distant | 96 | 85 | 71 | 59 | 0 | 1 | 1 | 1 | 4 | 14 | 28 | 40 |
|  | Unknown | 58 | 40 | 28 | 21 | 2 | 2 | 2 | 2 | 40 | 58 | 70 | 77 |

1. Cancer deaths are those due to the primary diagnosed-cancer and other causes include all other causes of death. Refer to text for further details.
2. Estimated from flexible parametric relative survival models that included age and year at diagnosis (both as continuous variables), interactions between age and year, and case mix for all solid cancers. Models stratified by sex (where relevant)
3. Models also included spread of disease and interactions between year of diagnosis and spread of disease

**Supplemental Table S2** Predicted crude probabilities of cancer, other cause deaths and being alive (per 100 persons), at 10 years after diagnosis by sex, spread of disease and period, 1985-2014, at age 65 years.

| ***Crude probabilities (Of 100 cancer patients, how many would have each status at 10-years follow-up)^1^*** | | | | | | | | | | | | | |
| --- | --- | --- | --- | --- | --- | --- | --- | --- | --- | --- | --- | --- | --- |
|  |  | Death due to cancer | | | | Death from other causes | | | | Alive | | | |
| Cancer | Spread of disease | *Year of diagnosis* | | | | *Year of diagnosis* | | | | *Year of diagnosis* | | | |
|  |  | *1985* | *1995* | *2005* | *2014* | *1985* | *1995* | *2005* | *2014* | *1985* | *1995* | *2005* | *2014* |
| ***MALES*** | | | | | | | | | | | | | |
| Colorectal | Overall^2^ | 49 | 41 | 35 | 32 | 17 | 15 | 12 | 10 | 34 | 44 | 53 | 58 |
|  | Localised^3^ | 18 | 13 | 12 | 14 | 26 | 21 | 15 | 12 | 56 | 66 | 73 | 74 |
|  | Regional | 49 | 38 | 36 | 40 | 19 | 16 | 12 | 10 | 32 | 46 | 52 | 50 |
|  | Distant | 95 | 90 | 88 | 91 | 3 | 4 | 4 | 3 | 2 | 6 | 8 | 6 |
|  | Unknown | 43 | 33 | 30 | 33 | 19 | 17 | 12 | 10 | 38 | 50 | 58 | 57 |
| Lung | Overall | 90 | 88 | 86 | 84 | 5 | 4 | 3 | 3 | 5 | 8 | 11 | 13 |
|  | Localised | 75 | 72 | 71 | 72 | 10 | 8 | 6 | 5 | 15 | 20 | 23 | 23 |
|  | Regional | 88 | 85 | 85 | 86 | 6 | 5 | 4 | 4 | 6 | 10 | 11 | 10 |
|  | Distant | 96 | 96 | 96 | 96 | 2 | 1 | 1 | 1 | 2 | 3 | 3 | 3 |
|  | Unknown | 89 | 88 | 87 | 88 | 5 | 4 | 3 | 3 | 6 | 8 | 10 | 9 |
| Melanoma | Overall | 20 | 14 | 11 | 11 | 25 | 20 | 15 | 12 | 55 | 66 | 74 | 77 |
|  | Localised | 6 | 6 | 6 | 6 | 29 | 22 | 16 | 13 | 65 | 72 | 78 | 81 |
|  | Regional | 43 | 41 | 41 | 43 | 20 | 16 | 11 | 9 | 37 | 43 | 48 | 48 |
|  | Distant | 75 | 73 | 73 | 75 | 9 | 7 | 5 | 4 | 16 | 20 | 22 | 21 |
|  | Unknown | 19 | 18 | 18 | 19 | 26 | 20 | 14 | 12 | 55 | 62 | 68 | 69 |
| Prostate | Overall | 56 | 15 | 3 | 1 | 17 | 21 | 16 | 13 | 27 | 64 | 81 | 86 |
|  | Localised | 12 | 9 | 6 | 3 | 28 | 22 | 16 | 13 | 60 | 69 | 78 | 84 |
|  | Regional | 35 | 27 | 18 | 11 | 24 | 19 | 15 | 13 | 41 | 54 | 67 | 76 |
|  | Distant | 91 | 85 | 71 | 51 | 6 | 7 | 7 | 8 | 3 | 8 | 22 | 41 |
|  | Unknown | 25 | 19 | 13 | 7 | 25 | 20 | 15 | 13 | 50 | 61 | 72 | 80 |
| All solid | Overall | 65 | 45 | 31 | 23 | 13 | 14 | 12 | 11 | 22 | 41 | 57 | 66 |
|  | Localised | 23 | 19 | 17 | 15 | 25 | 19 | 14 | 12 | 52 | 62 | 69 | 73 |
|  | Regional | 58 | 51 | 46 | 42 | 15 | 13 | 10 | 9 | 27 | 36 | 44 | 49 |
|  | Distant | 93 | 91 | 88 | 84 | 3 | 3 | 3 | 3 | 4 | 6 | 9 | 13 |
|  | Unknown | 39 | 34 | 30 | 27 | 20 | 16 | 12 | 10 | 41 | 50 | 58 | 63 |
| ***FEMALES*** | | | | | | | | | | | | | |
| Colorectal | Overall | 47 | 39 | 32 | 28 | 10 | 9 | 8 | 7 | 43 | 52 | 60 | 65 |
|  | Localised | 19 | 13 | 8 | 5 | 15 | 12 | 10 | 8 | 66 | 75 | 82 | 87 |
|  | Regional | 57 | 42 | 29 | 19 | 9 | 9 | 8 | 7 | 34 | 49 | 63 | 74 |
|  | Distant | 97 | 93 | 82 | 66 | 1 | 2 | 3 | 4 | 2 | 5 | 15 | 30 |
|  | Unknown | 52 | 38 | 26 | 17 | 9 | 9 | 8 | 7 | 39 | 53 | 66 | 76 |
| Lung | Overall | 90 | 87 | 83 | 76 | 3 | 3 | 3 | 3 | 7 | 10 | 14 | 21 |
|  | Localised | 67 | 63 | 60 | 60 | 7 | 6 | 5 | 4 | 26 | 31 | 35 | 36 |
|  | Regional | 87 | 85 | 83 | 82 | 4 | 3 | 3 | 3 | 9 | 12 | 14 | 15 |
|  | Distant | 97 | 96 | 96 | 95 | 1 | 1 | 1 | 1 | 2 | 3 | 3 | 4 |
|  | Unknown | 88 | 85 | 84 | 83 | 3 | 3 | 3 | 2 | 9 | 12 | 13 | 15 |
| Melanoma | Overall | 13 | 9 | 7 | 5 | 16 | 12 | 10 | 8 | 71 | 79 | 83 | 87 |
|  | Localised | 4 | 3 | 3 | 3 | 17 | 13 | 10 | 8 | 79 | 84 | 87 | 89 |
|  | Regional | 43 | 37 | 35 | 38 | 12 | 9 | 8 | 6 | 45 | 54 | 57 | 56 |
|  | Distant | 80 | 73 | 71 | 74 | 5 | 4 | 4 | 3 | 15 | 23 | 25 | 23 |
|  | Unknown | 14 | 12 | 11 | 12 | 16 | 12 | 10 | 8 | 70 | 76 | 79 | 80 |
| Breast | Overall | 37 | 19 | 12 | 11 | 13 | 11 | 9 | 8 | 50 | 70 | 79 | 81 |
|  | Localised | 9 | 6 | 5 | 4 | 16 | 12 | 10 | 8 | 75 | 82 | 85 | 88 |
|  | Regional | 39 | 27 | 21 | 19 | 13 | 11 | 9 | 7 | 48 | 62 | 70 | 74 |
|  | Distant | 87 | 73 | 63 | 60 | 4 | 5 | 5 | 5 | 9 | 22 | 32 | 35 |
|  | Unknown | 42 | 29 | 22 | 21 | 12 | 10 | 9 | 7 | 46 | 61 | 69 | 72 |
| All solid | Overall | 53 | 41 | 33 | 29 | 9 | 8 | 7 | 6 | 38 | 51 | 60 | 65 |
|  | Localised | 27 | 18 | 13 | 10 | 14 | 11 | 9 | 8 | 59 | 71 | 78 | 82 |
|  | Regional | 64 | 48 | 36 | 28 | 8 | 8 | 7 | 7 | 28 | 44 | 57 | 65 |
|  | Distant | 97 | 92 | 83 | 74 | 1 | 2 | 3 | 3 | 2 | 6 | 14 | 23 |
|  | Unknown | 67 | 51 | 38 | 30 | 7 | 7 | 7 | 6 | 26 | 42 | 55 | 64 |

1. Cancer deaths are those due to the primary diagnosed-cancer and other causes include all other causes of death. Refer to text for further details.
2. Estimated from flexible parametric relative survival models that included age and year at diagnosis (both as continuous variables), interactions between age and year, and case mix for all solid cancers. Models stratified by sex (where relevant)
3. Models also included spread of disease and interactions between year of diagnosis and spread of disease

**Supplemental Table S3** Predicted crude probabilities of cancer, other cause deaths and being alive (per 100 persons), at 10 years after diagnosis by sex, spread of disease and period, 1985-2014, at age 80 years.

| ***Crude probabilities (Of 100 cancer patients, how many would have each status at 10-years follow-up)^1^*** | | | | | | | | | | | | | |
| --- | --- | --- | --- | --- | --- | --- | --- | --- | --- | --- | --- | --- | --- |
|  |  | Death due to cancer | | | | Death from other causes | | | | Alive | | | |
| Cancer | Spread of disease | *Year of diagnosis* | | | | *Year of diagnosis* | | | | *Year of diagnosis* | | | |
|  |  | *1985* | *1995* | *2005* | *2014* | *1985* | *1995* | *2005* | *2014* | *1985* | *1995* | *2005* | *2014* |
| ***MALES*** | | | | | | | | | | | | | |
| Colorectal | Overall^2^ | 37 | 35 | 33 | 30 | 50 | 47 | 44 | 40 | 13 | 18 | 23 | 30 |
|  | Localised^3^ | 13 | 12 | 12 | 13 | 70 | 65 | 58 | 50 | 17 | 23 | 30 | 37 |
|  | Regional | 36 | 34 | 35 | 37 | 53 | 50 | 45 | 39 | 11 | 16 | 20 | 24 |
|  | Distant | 88 | 87 | 87 | 88 | 9 | 10 | 10 | 9 | 3 | 3 | 3 | 3 |
|  | Unknown | 34 | 31 | 31 | 33 | 52 | 50 | 45 | 40 | 14 | 19 | 24 | 27 |
| Lung | Overall | 88 | 87 | 87 | 87 | 10 | 10 | 9 | 9 | 2 | 3 | 4 | 4 |
|  | Localised | 75 | 73 | 74 | 76 | 22 | 21 | 19 | 17 | 3 | 6 | 7 | 7 |
|  | Regional | 85 | 85 | 86 | 87 | 13 | 13 | 11 | 10 | 2 | 2 | 3 | 3 |
|  | Distant | 94 | 95 | 95 | 96 | 3 | 3 | 3 | 3 | 3 | 2 | 2 | 1 |
|  | Unknown | 86 | 86 | 87 | 88 | 11 | 11 | 9 | 8 | 3 | 3 | 4 | 4 |
| Melanoma | Overall | 21 | 16 | 14 | 14 | 65 | 63 | 57 | 50 | 14 | 21 | 29 | 36 |
|  | Localised | 6 | 5 | 6 | 8 | 76 | 70 | 62 | 53 | 18 | 25 | 32 | 39 |
|  | Regional | 42 | 39 | 43 | 51 | 50 | 48 | 41 | 33 | 8 | 13 | 16 | 16 |
|  | Distant | 78 | 75 | 77 | 84 | 19 | 20 | 17 | 12 | 3 | 5 | 6 | 4 |
|  | Unknown | 18 | 17 | 18 | 23 | 68 | 63 | 56 | 47 | 14 | 20 | 26 | 30 |
| Prostate | Overall | 41 | 26 | 15 | 8 | 51 | 57 | 57 | 53 | 8 | 17 | 28 | 39 |
|  | Localised | 9 | 7 | 5 | 3 | 74 | 69 | 63 | 55 | 17 | 24 | 32 | 42 |
|  | Regional | 27 | 22 | 16 | 10 | 62 | 60 | 57 | 53 | 11 | 18 | 27 | 37 |
|  | Distant | 85 | 79 | 69 | 51 | 13 | 18 | 24 | 31 | 2 | 3 | 7 | 18 |
|  | Unknown | 20 | 16 | 11 | 7 | 67 | 64 | 59 | 54 | 13 | 20 | 30 | 39 |
| All solid | Overall | 53 | 46 | 42 | 39 | 39 | 40 | 39 | 36 | 8 | 14 | 19 | 25 |
|  | Localised | 16 | 19 | 20 | 20 | 68 | 61 | 53 | 47 | 16 | 20 | 27 | 33 |
|  | Regional | 45 | 49 | 53 | 53 | 46 | 39 | 33 | 30 | 9 | 12 | 14 | 17 |
|  | Distant | 86 | 89 | 91 | 91 | 11 | 8 | 6 | 6 | 3 | 3 | 3 | 3 |
|  | Unknown | 30 | 33 | 36 | 36 | 57 | 50 | 43 | 38 | 13 | 17 | 21 | 26 |
| ***FEMALES*** | | | | | | | | | | | | | |
| Colorectal | Overall | 45 | 39 | 35 | 32 | 36 | 36 | 34 | 32 | 19 | 25 | 31 | 36 |
|  | Localised | 16 | 13 | 10 | 8 | 55 | 51 | 47 | 43 | 29 | 36 | 43 | 49 |
|  | Regional | 52 | 43 | 35 | 27 | 34 | 35 | 35 | 35 | 14 | 22 | 30 | 38 |
|  | Distant | 95 | 92 | 88 | 80 | 3 | 4 | 7 | 11 | 2 | 4 | 5 | 9 |
|  | Unknown | 50 | 41 | 32 | 25 | 32 | 34 | 35 | 35 | 18 | 25 | 33 | 40 |
| Lung | Overall | 88 | 88 | 88 | 84 | 8 | 8 | 8 | 9 | 4 | 4 | 4 | 7 |
|  | Localised | 67 | 67 | 68 | 70 | 24 | 22 | 19 | 17 | 9 | 11 | 13 | 13 |
|  | Regional | 86 | 86 | 87 | 88 | 11 | 11 | 9 | 9 | 3 | 3 | 4 | 3 |
|  | Distant | 96 | 96 | 96 | 96 | 3 | 3 | 2 | 2 | 1 | 1 | 2 | 2 |
|  | Unknown | 86 | 87 | 88 | 88 | 10 | 9 | 8 | 7 | 4 | 4 | 4 | 5 |
| Melanoma | Overall | 15 | 14 | 11 | 6 | 57 | 51 | 47 | 43 | 28 | 35 | 42 | 51 |
|  | Localised | 4 | 3 | 3 | 3 | 64 | 57 | 50 | 44 | 32 | 40 | 47 | 53 |
|  | Regional | 42 | 40 | 39 | 39 | 42 | 38 | 34 | 32 | 16 | 22 | 27 | 29 |
|  | Distant | 80 | 79 | 78 | 77 | 14 | 14 | 13 | 14 | 6 | 7 | 9 | 9 |
|  | Unknown | 14 | 13 | 13 | 13 | 58 | 52 | 46 | 42 | 28 | 35 | 41 | 45 |
| Breast | Overall | 28 | 22 | 19 | 19 | 50 | 48 | 43 | 39 | 22 | 30 | 38 | 42 |
|  | Localised | 7 | 7 | 6 | 6 | 62 | 55 | 49 | 44 | 31 | 38 | 45 | 50 |
|  | Regional | 30 | 29 | 28 | 26 | 49 | 44 | 40 | 37 | 21 | 27 | 32 | 37 |
|  | Distant | 79 | 77 | 75 | 71 | 15 | 14 | 14 | 17 | 6 | 9 | 11 | 12 |
|  | Unknown | 33 | 33 | 31 | 28 | 46 | 42 | 38 | 36 | 21 | 25 | 31 | 36 |
| All solid | Overall | 54 | 52 | 49 | 44 | 31 | 29 | 28 | 27 | 15 | 19 | 23 | 29 |
|  | Localised | 26 | 22 | 18 | 14 | 50 | 47 | 43 | 40 | 24 | 31 | 39 | 46 |
|  | Regional | 61 | 54 | 46 | 38 | 28 | 30 | 30 | 31 | 11 | 16 | 24 | 31 |
|  | Distant | 95 | 93 | 91 | 85 | 3 | 4 | 5 | 8 | 2 | 3 | 4 | 7 |
|  | Unknown | 67 | 59 | 50 | 41 | 22 | 25 | 26 | 28 | 11 | 16 | 24 | 31 |

1. Cancer deaths are those due to the primary diagnosed-cancer and other causes include all other causes of death. Refer to text for further details.
2. Estimated from flexible parametric relative survival models that included age and year at diagnosis (both as continuous variables), interactions between age and year, and case mix for all solid cancers. Models stratified by sex (where relevant)
3. Models also included spread of disease and interactions between year of diagnosis and spread of disease

Supplemental Figure 1: Temporal trends in the percentage of cases with unknown spread of disease by sex and major cancer types, NSW, Australia, 1985-2014


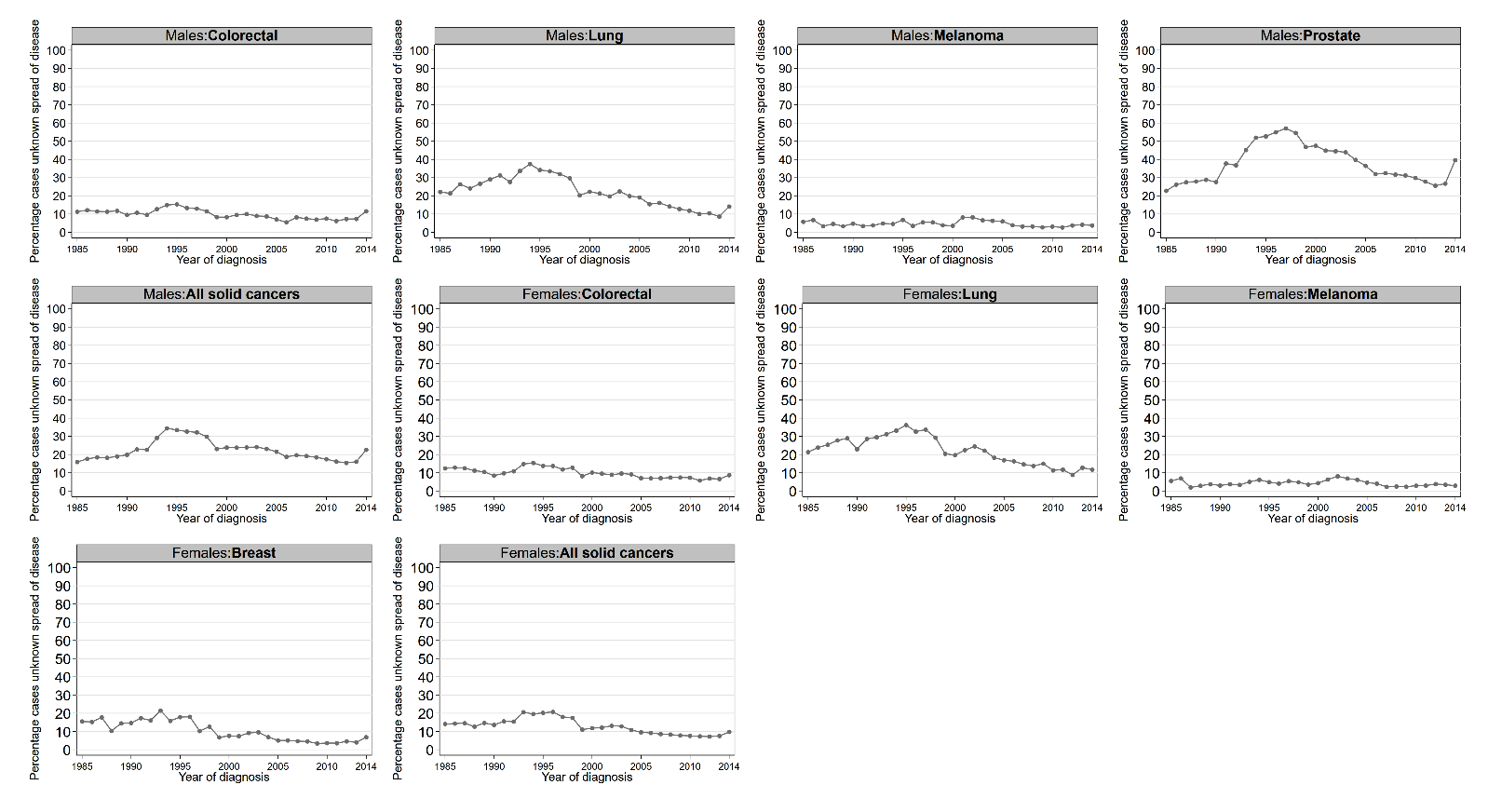


Supplemental Figure 2: Temporal trends in the predicted 10-year crude probability of death due to cancer (solid lines) and other causes (dashed lines) for prostate cancer and female lung cancer at selected ages with different assumptions (A to D) for unknown spread of disease at diagnosis NSW, Australia, 1985-2014


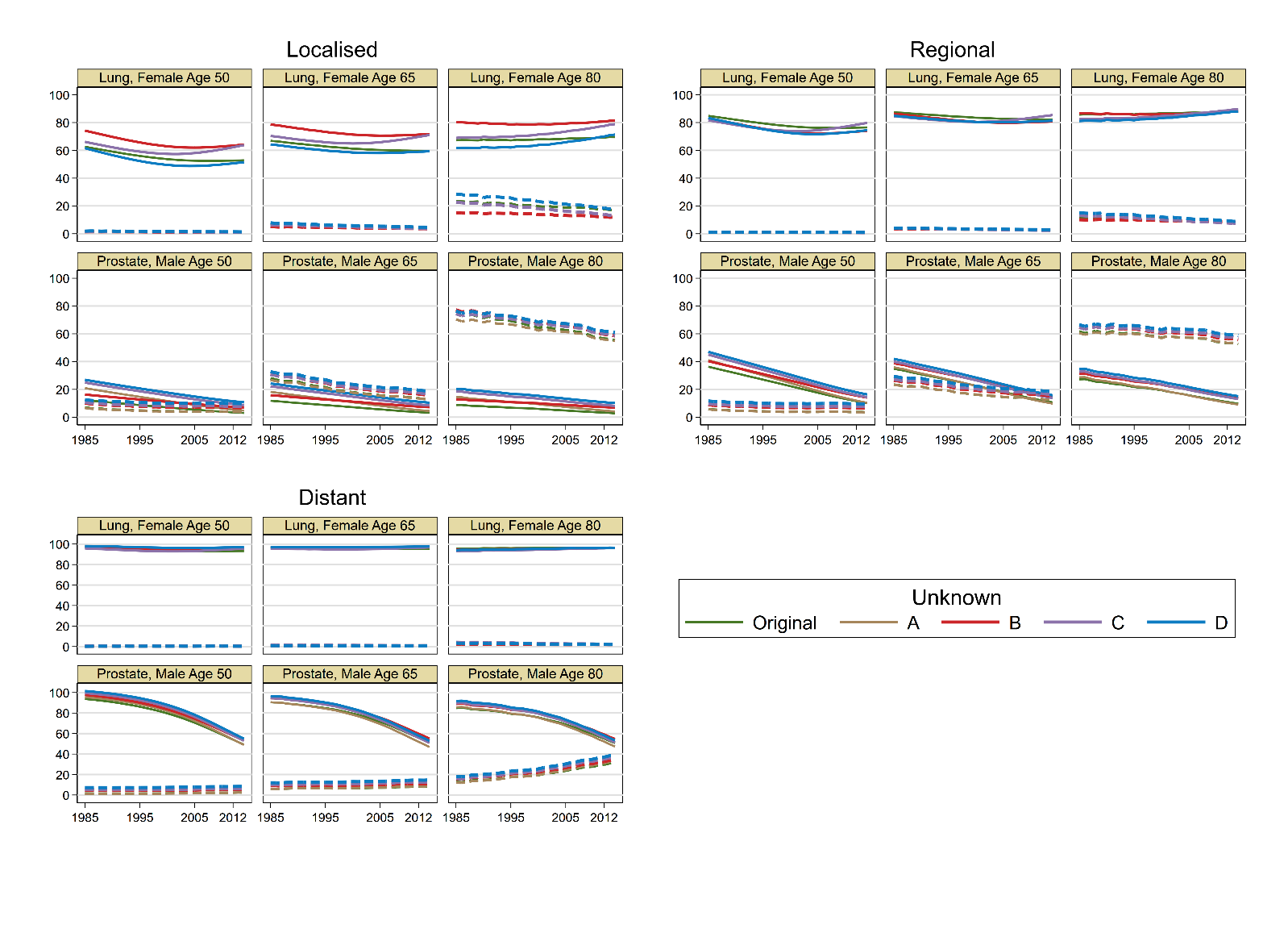


*Assumptions were that all cases with unknown spread of disease were: A localised, B distant, C randomly and equally distributed over three categories (localised, regional and distant), and D assigned on the basis of the survival experience. The x axis in each graph is the ‘Year of Diagnosis’ and the y axis the ‘Crude probability of death’. Dark green represents estimates for original cohort, gold (Assumption A), red (Assumption B), violet (Assumption C) and light blue (Assumption D).*
